# Comparative analysis of copy number variation detection methods and database construction

**DOI:** 10.1186/1471-2156-12-29

**Published:** 2011-03-07

**Authors:** Asako Koike, Nao Nishida, Daiki Yamashita, Katsushi Tokunaga

**Affiliations:** 1 Central Research Laboratory, Hitachi Ltd. Tokyo, Japan; 2 Department of Human Genetics, Graduate School of Medicine, University of Tokyo, Tokyo, Japan

## Abstract

**Background:**

Array-based detection of copy number variations (CNVs) is widely used for identifying disease-specific genetic variations. However, the accuracy of CNV detection is not sufficient and results differ depending on the detection programs used and their parameters. In this study, we evaluated five widely used CNV detection programs, Birdsuite (mainly consisting of the Birdseye and Canary modules), Birdseye (part of Birdsuite), PennCNV, CGHseg, and DNAcopy from the viewpoint of performance on the Affymetrix platform using HapMap data and other experimental data. Furthermore, we identified CNVs of 180 healthy Japanese individuals using parameters that showed the best performance in the HapMap data and investigated their characteristics.

**Results:**

The results indicate that Hidden Markov model-based programs PennCNV and Birdseye (part of Birdsuite), or Birdsuite show better detection performance than other programs when the high reproducibility rates of the same individuals and the low Mendelian inconsistencies are considered. Furthermore, when rates of overlap with other experimental results were taken into account, Birdsuite showed the best performance from the view point of sensitivity but was expected to include many false negatives and some false positives. The results of 180 healthy Japanese demonstrate that the ratio containing repeat sequences, not only segmental repeats but also long interspersed nuclear element (LINE) sequences both in the start and end regions of the CNVs, is higher in CNVs that are commonly detected among multiple individuals than that in randomly selected regions, and the conservation score based on primates is lower in these regions than in randomly selected regions. Similar tendencies were observed in HapMap data and other experimental data.

**Conclusions:**

Our results suggest that not only segmental repeats but also interspersed repeats, especially LINE sequences, are deeply involved in CNVs, particularly in common CNV formations.

The detected CNVs are stored in the CNV repository database newly constructed by the "Japanese integrated database project" for sharing data among researchers. http://gwas.lifesciencedb.jp/cgi-bin/cnvdb/cnv_top.cgi

## Background

Copy number variations (CNVs), duplications, and deletions of chromosomal segments longer than 1 kb are major structural variations in various organisms such as yeast, Drosophila, and humans and are believed to have evolutionary importance [1] and be related to various diseases such as mental retardation [2], neurological disorders [3,4], and cancers [5]. Although chromosomal abnormalities including CNVs were originally investigated using cytogenetic karyotype analyses such as chromosome banding analysis and fluorescence in situ hybridization (FISH), recent technologies such as pair-end sequencing [6-8], whole genome sequencing using massively parallel sequencers [9], and array-based approaches such as array comparative genomic hybridization (CGH) [10] and single nucleotide polymorphism (SNP) genotyping microarrays [11]) enabled us to obtain refined CNV structures with relatively high throughput. Particularly, SNP genotyping microarrays are becoming widely used for CNV identification since CNVs can be detected using the same microarrays used in the SNP-based case control study. The SNP microarray approaches, however, tend to lead to too many false negatives and positives and require sophisticated CNV detection methods/algorithms.

Many methods/algorithms have been proposed for detecting CNVs for array CGH and SNP array and are divided into several models such as smoothing methods, clustering methods, maximum likelihood procedures including Hidden Markov models (HMMs) and expectation-maximization (EM) algorithms. The simplest smoothing method is for smoothing log_2_ratio (ratio is defined as the intensity of target of probe divided by that of reference probe) profiles using a moving average and detecting duplicated or deleted regions over the specified thresholds [12]. For more sophisticated smoothing methods, a quantile smoothing method based on L1 norm (the sum of absolute values) penalty minimization [13] and a wavelet de-noising method [14] have been proposed. For a clustering method, the cluster along with chromosomes (CLAC) method was developed, in which hierarchical clustering trees along each chromosome arm (or chromosome) are calculated and the 'interesting' clusters considering the false discovery rate (FDR) are selected [15]. Smoothing and clustering methods are effective in simulation data, but they do not achieve good enough CNV detection performance compared with other methods in array CGH experimental data [16]. To date, various maximum likelihood-related approaches have been proposed. Jong *et al. *introduced genetic local search algorithms (memetic algorithms) for maximizing the likelihood by considering the penalty function of breakpoints [17]. Picard *et al. *developed an adaptive method for estimating the penalty constant to avoid selecting too large a segmentation number for over fitting given data. In this method, the probe intensity profile (log_2_ratio) is supposed to be a Gaussian distribution, and the number of segments is estimated by maximizing the likelihood [18]. A circular binary segmentation (CBS) method was proposed by Venkatraman and Olshen [19], in which the average probe intensity is assumed to also have a Gaussian distribution. The likelihood ratio statistic for testing the null hypothesis, in which there is no change, and the alternative hypothesis, in which there is exactly one change at an unknown location, are introduced in this method. The test is done using a permutation test. If the null hypothesis is rejected, the hypothetical change-points are adopted. The change-points are searched recursively using overlapping windows [19].

An HMM is a statistical model in which it is assumed that the system follows a Markov process [20-22]. In most HMM models for CNV detection methods, the probe intensity values or logR ratio (LRR, log_2_(R_observed_/R_expected_), R_expected _is calculated from linear interpolation of canonical genotype clusters, R is a sum of probe intensities) and B allele frequency (BAF, normalized measure of relative signal intensity ratio of the B and A alleles in the SNP array) or genotypes are assumed to be independent, and the copy number states of the probes are set to be hidden states with certain transition probabilities. By maximizing the likelihood of observed data (probe intensity, LRR and BAF, or genotypes), the copy number state of each probe is obtained.

Many studies have compared the performance of these methods or programs based mainly on simulation data, arrayCGH data [16] and Illumina SNP arrays [23]. However, Affymetrix SNP-array-based CNV detection requires much more robust algorithms than those using Illumina SNP arrays and array CGH due to the characteristics of the Affymetrix SNP arrays.

We assessed the following widely used methods/programs, the circular binary segmentation (CBS) method (implemented in DNAcopy [19], R package), Picard's adaptive method (CGHseg [18], R package), HMMs (Birdsuite 1.4 [20] and PennCNV [21]) on the basis of whether they accurately detect CNVs on Affymetrix data (Affymetrix 6.0). The first two methods/programs are known to be effective in detecting CNVs of array-CGH experiments, but their performances in microarrays are yet unknown. QuantiSNP [22], which uses an objective Bayes HMM, showed the best detection performance with the simulation and Illumina SNP array data in a previous study [23]. We also tested QuantiSNP, but the parameter tunings for Affymetrix data were too difficult for us to achieve sufficient performance. Therefore, the results are not shown in the following results. The assessments are done using HapMap data and other experimental results. After that, CNVs of 180 healthy Japanese individuals were detected using the parameters that showed the best performance in the HapMap data. The characteristics of start and end regions of CNVs are also discussed. These results are registered in the CNV control database, which has been developed as part of the Japanese integrated database project.

## Methods

### CNV detection methods

We conducted comparative analyses on the CNV detection performance of five programs: DNAcopy [19] (R package) as a circular binary segmentation (CBS) method, CGHseg [18] ("tiling array" of R) as a Picard's adaptive method, Birdseye (part of Birdsuite) [20] and PennCNV [21] as HMMs, and Birdsuite (mainly consisting of Birdseye and the EM-based Canary).

There are two main subjects for CNV detection: estimation of the copy numbers and detection of accurate CNV boundaries from probe intensity data. In this study, duplications (gains) or deletions (losses) were assessed, but copy numbers were not assessed because there were not enough validation data to confirm them. In the programs for array CGH, the log_2_ratios of normalized intensities of target probes against control probes were used as input data, while programs for the SNP arrays, not only the log_2_ratios or LRR but also allele frequencies or genotypes were also used in most cases. Since DNAcopy and CGHseg were developed for array CGH, only log_2_ratios were used as input data. During log_2_ratio calculation, quantile-normalization was done for probe intensities and the average values of HapMap data (270 individuals) were used as reference probe intensity data.

Birdsuite consists of four programs (modules), "Canary", "Birdseed", "Birdseye", and "Fawkes," and determines CNVs using multiple individual data. In Canary, common CNVs are detected with EM algorithms using registered known common copy number data, while Birdseye detects novel CNVs by using the HMM with the Viterbi algorithm based on probe intensities and genotypes determined using the Birdseed program. Fawkes combines results of Canary, Birdseye, and Birdseed and assigns a comprehensive SNP genotype. Since we do not access the genotypes of CNVs, we used only Canary and Birdseye to obtain the following results.

In PennCNV, probe intensity data is converted into LRR and BAF, copy numbers are set as hidden states, and the emission probability of LRR and BAF are modeled. The hidden state for maximizing the likelihood of observed data (LRR and BAF) is obtained using the Viterbi algorithm.

### Data sets

To compare the detection performance of these algorithms, we used Affymetrix Genome-wide Human SNP Array 6.0 (Affy 6.0) data of the HapMap data. The HapMap data were collected from 45 Japanese in Tokyo, Japan, 45 Han Chinese in Beijing, China, 90 Yoruba individuals (30 trios) in Ibadan, Nigeria, and 90 individuals from the US state of Utah with northern and western European ancestry (collected in 1980 by the Centre d'Etude du Polymorphisme Humain) whose CNVs were previously measured in other experiments and/or algorithms [6-8,11,20,21,24-29]. Furthermore, five sets of five Affy 6.0 microarrays of the same individuals (NA04626, NA01416, NA06061, NA10851, NA15510) were also used to investigate the reproducibility of the detection algorithm. These Affy 6.0 data were typed in Affymetrix and downloaded from the Affymetrix web site [30].

Furthermore, novel Affy 6.0 data of 180 healthy Japanese individuals, whose typing was carried out in our previous study [31], were used for detecting Japanese CNVs. (Hereinafter we call these data "original data.") As described in the previous paper [31], the study was approved by the research ethics committee of Central Research Laboratory, Hitachi Ltd. (permission number 128-2) and the Faculty of Medicine, The University of Tokyo (permission number 2583) and the informed consent was obtained from all participants.

### Database construction

A CNV control database has been constructed as part of the integrated database project by the Ministry of Education, Culture, Sports, Science and Technology (MEXT) using mySQL. In the database, the start-end information of copy number segments, copy number regions which are clustered copy number segment information, and their frequencies were accumulated. Other information such as genes, exons, introns, and the CNVs of database of genomic variants (DGV [32]) data were also accumulated to enable users to easily interpret the meaning of detected CNV regions. The start and end positions of the CNVs were also stored on a distributed annotation system (DAS) with the GMOD Gbrowse-based [33] browser for calling up our data on other DAS servers, such as Ensemble, or vice versa; the data on another DAS are called up on our DAS to view various data simultaneously for interpretation.

## Results and discussion

### Evaluation of CNV detection algorithms using trios data

To evaluate the CNV detection performance of each method, the concordance of CNV detections was investigated using five sets of microarrays for the same individuals (five microarrays per individual×five individuals). Table 1 summarizes the average stability rates of CNVs at the segment level to measure CNV reproducibility using different DNA from the same individual. (Analyses were done by copy number segments if there are no explanations.) Copy number segments with overlap > 80% were regarded as concordant segments. Even if the overlap threshold was changed (20-80%), this tendency was almost the same. In this stability rate, duplications and deletions were discriminated, but copy number differences within duplications or deletions were not distinguished. The CNV detection thresholds (parameters) in each method were determined to maximize the stability rate. These thresholds show the best performance (minimum value) also in the Mendelian inconsistency ratio of trio data (Table 2). Furthermore, when other array data for the same individuals are used as reference samples instead of the average of HapMap data and these thresholds are applied, neither DNAcopy nor CGHseg show false positives (that is, CNVs are not detected).

**Table 1:** Average stability rate of CNVs using same individual's data

Programs	Average of concordance rate
PennCNV	89.4%
Birdsuite:Birdseye/Canary	91.1/94.6%
DNAcopy	82.0%
CGHseg	79.6%

Stability rate is the concordance rate of CNVs detected using different DNA samples from the same person. Segment overlap > 80% is regarded as concordant.

All versus all comparisons (5 microarray analysis×5 microarray analysis) were done.

PennCNV: Threshold of number of probes > 20

Birdseye (part of Birdsuite): Threshold of number of probes > 20 and Lod score > 5

DNAcopy: Threshold of number of probes > 30 and absolute avg. intensity of a segment > 0.35

CGHseg: Threshold of number of probes > 40 and absolute avg. intensity of a segment > 0.35

**Table 2:** The rate of Mendelian inconsistencies in offsprings' CNVs of HapMap trio data

Programs	Overlap > 0%	> 50%	> 80%
PennCNV	0.190	0.192	0.197
Birdsuite:Birdseye/Canary	0.028/0.015	0.030/0.015	0.030/0.015
DNAcopy	0.531	0.579	0.583
CGHseg	0.417	0.462	0.493

The stability rate does not directly predict performance but indicates the stability of detection performance. Table 1 shows that PennCNV and Birdseye (part of Birdsuite) have the same level of detection stability, about 10% higher than those of DNA copy and CGHseg, while Canary (part of Birdsuite) achieves higher stability than others due to the utilization of pre-defined copy number regions.

Table 2 shows the rate of Mendelian inconsistencies of HapMap trio (90 individuals) data. The rate is calculated as the average number of an offspring's CNVs that are not detected in both parents divided by the number of each offspring's CNVs. Although a rate of Mendelian inconsistencies close to 0 does not directly mean high CNV detection performance, the rate of Mendelian inconsistencies much larger than 0 implies low CNV detection performance considering heritability because the frequency of copy number change is assumed to be in the range of 10^-6 ^to 10^-4 ^per gamete [24,34]. The rates of Mendelian inconsistencies of Canary (part of Birdsuite), and Birdseye (part of Birdsuite) in Table 2 are the lowest and second lowest, respectively; however, there may be influences of the parameters which are tuned using HapMap data [20] considering the low stability rate of Birdseye in Table 1 (that is, the square-root of the stability rate << 1-Mendelian inconsistency in Birdseye). For Canary, since only predefined regions are the targets of CNV detection, low Mendelian inconsistencies of Canary are reasonable. The low Mendelian inconsistency is a necessary but not sufficient condition for high accuracy for CNV detections. Mendelian inconsistencies of all methods, except Canary and Birdseye, are not sufficiently low, and DNAcopy shows the highest Mendelian inconsistencies, although the stability rate in Table 1 is the same level as CGHseg. The cause is not clear, but there might be CNV patterns that are difficult to detect with circular binary-segmentation-based detection for DNAcopy.

**Figure 1:**
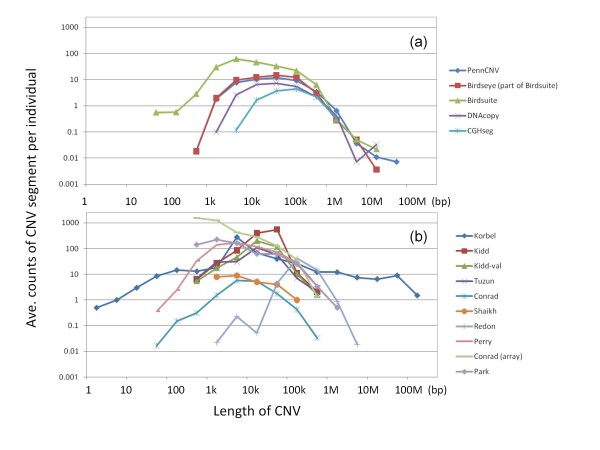
**Distribution of CNV lengths per individual for each experiment**.

The similarity of CNVs detected using each program is measured as the sensitivity between programs in Table 3. Although PennCNV and Birdseye (part of Birdsuite) use a HMM, similarity between them is not so large in all (deletions + duplications) CNV data. The difference between them might be partly caused by parameter settings. Overall, deletions are higher than all (deletions + duplications) CNVs. DNAcopy shows a comparatively high similarity compared with all other programs considering the small number of CNVs detected by DNAcopy. The dependence of the similarity on CNV length was not clearly observed. In Figure 1 (a), the distributions of CNV length using each program/method are depicted, as discussed later.

**Table 3:** Similarity of CNVs detected between programs using HapMap data

Programs	PennCNV	Birdseye (part of Birdsuite) Birdsuite	DNAcopy	CGHseg
PennCNV [12417/6895]	-	55.1/62.2* (56.4/61.9)	62.5/75.2 (63.1/76.7)	49.2/63.5 (51.5/65.3)
		23.2/26.8 (23.8/26.9)		
Birdseye (part of Birdsuite) [14794/10102]	62.2/69.8 (82.8/88.5)	-	57.5/68.4 (69.8/82.6)	40.7/52.4 (49.1/61.6)
Birdsuite [56476/44150]	68.0/74.3 (90.3/93.3)		68.8/73.9 (84.5/89.4)	47.0/54.4 (57.5/63.3)
DNAcopy [6748/4849]	37.7/40.8 (50.7/53.8)	29.6/31.8 (38.0/40.4)	-	61.1/68.6 (62.1/68.6)
		13.8/15.0 (16.4/17.7)		
CGHseg [3648/2401]	13.8/17.4 (17.1/20.9)	10.1/12.3 (11.6/13.9)	32.1/35.6 (27.4/32.0)	-
		4.2/5.2 (4.6/5.6)		

Similarity: percentage of CNVs detected using the program in row, which is also detected using the program in column. That is, each program in row is regard as golden standard.

*Sensitivity with 80%overlap/5%overlap of CNV segments

(): represents deletion-only data

[]: represents total number of CNVs/total number of CNV deletions.

**Figure 2:**
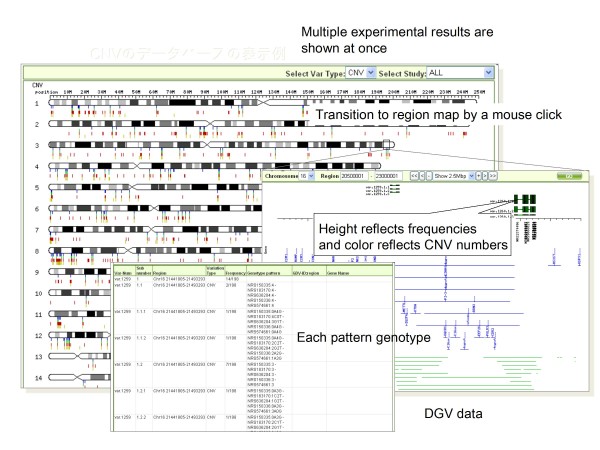
**Screen shots of CNV control database**. Screenshot of overview of CNVs, region information, and CNV start and end information of CNVs in CNV control database.

Affymetrix data is known to be noisy for determining genotypes under some experimental conditions. When detecting CNVs, data noise is expected to be problematic. Supplemental Figures 1S-a (HapMap) and 2S-a (180 healthy Japanese individuals) [Additional file 1: Supplemental Figures 1S and 2S] show the relationship between the standard deviation of log_2_ratio in each array and CNV counts per individual, while supplemental Figures 1S-b (HapMap) and 2S-b (180 healthy Japanese individuals) [Additional file 1: Supplemental figures 1S and 2S] show the relationships between the call rate (the percentage of SNPs whose genotypes are determined in the genotype calling process) and CNV counts per individual detected using each program. Figures 1S-a and 2S-a indicate that the CNV counts per individual drastically increase when the standard deviation of the log_2_ratio of the array is high. Figures 1S-b and 2S-b indicate that Affymetrix arrays with low recall showed higher CNV counts. Since these tendencies were observed in all programs, arrays with large standard deviations or with low recall should be removed. In the analysis of original data in Section 'CNVs of original data of healthy Japanese', individuals with high CNV counts (15 individuals whose CNV number are PennCNV > 250, Birdseye (part of Birdsuite) > 400, Canary (part of Birdsuite) > 500, DNAcopy > 100, or CGHseg > 100) were removed.

### Evaluation of CNV detection algorithms by comparing other experimental results

The CNVs of these HapMap individuals have also been investigated using various experimental approaches [6-8,11,24-29], and a comparison of these previous studies is summarized in Table 4. In Table 4, the HapMap individuals used in both this study and in each experiment are used to calculate sensitivity. The corresponding specificity is summarized in supplemental Table 1S [Additional file 2: Supplemental Table 1S]. There are no tendencies of longer CNVs being commonly detected in other experiments (data not shown). The overlap among these experiments' CNVs is low, as shown in supplemental Table 2S [Additional file 2: Supplemental Table 2S]. The inconsistent results of various experiments also make it difficult to validate CNV detection methods. These experimental data probably include many false negatives and some false positives. The sensitivities become slightly higher when the detection thresholds are changed. However, considering the CNVs' reproducibility (stability) for the same individual, these thresholds seem plausible. In most cases, the sensitivity and specificity are high in "deletion-only data". This is partly because some of the experimental results do not distinguish "insertion" and "duplication". When the results of Birdseye (part of Birdsuite) and Birdsuite are compared, the sensitivities are increased but the specificities are largely decreased; because of that, the specificity of Canary (part of Birdsuite) is low. Since these other experiments are expected to include false negatives, low specificity may not directly mean low accuracy of the program. Given that the CNV detection's stability of the same individuals is high and the Mendelian inconsistency ratio is comparatively low in PennCNV, Birdseye (part of Birdsuite), and Birdsuite (mainly consisting of Birdseye and Canary), they are expected to show higher sensitivity and specificity than those of other programs. It should be noted that the sensitivities of Birdseye (part of Birdsuite), Birdsuite, and PennCNV in Table 4 are higher than those of DNAcopy and CGHseg in Conrad [28] and Park's [29] results, which are expected to be more reliable than earlier data such as Redon [11] and Korbel [6] when their estimated false discovery rate (Conrad *et al. *15% [28]) and Mendelian inconsistencies (Park *et al. *2.6% [29]) are considered, although the sensitivities of all programs are quite low in some experiments. Table 1S, however, implies that there are no striking differences among specificities of these CNV detection programs. This indicates that detection programs are expected to have many false negatives and a smaller number of false positives.

**Table 4:** Sensitivity of each detection program when other experimental result is regarded as golden standard

Programs	**Korbel**[6]**(PE sequencing)**	**Kidd**[7]**(PE sequencing)**	**Kidd **[7]**-validation**	**Tuzun**[8]**(PE sequencing)**	**Conrad**[25]**(Mendelian consistency)	**Shaikh**[26]** (Illumine Data)	**Redon**[11]**(Tiling array)**	**Perry**[27]**(array CGH)**	**Conrad**[28]**(Tiling array)**	**Park**[29]**(array-CGH and massively parallel sequencing)**
PennCNV	2.2* (2.1)	0.5 (0.7)	1.2 (1.5)	5.2 (7.8)	23.7	37.0 [37.1]	1.2 (1.1)	18.1 (22.3)	26.2 (24.5)	27.0 (33.0)
Birdseye (part of Birdsuite)	2.4 (2.4)	0.6 (1.0)	1.4 (2.1)	7.7 (13.9)	30.0	40.7 [44.4]	0.8 (0.9)	13.4 (14.0)	23.5 (24.1)	27.9 (36.9)
Birdsuite	5.5 (6.1)	2.0 (4.3)	5.5 (8.8)	17.2 (24.8)	46.1	51.9 [70.4]	2.2 (3.2)	25.6 (30.0)	35.4 (35.9)	42.9 (56.2)
DNAcopy	1.7 (1.6)	0.4 (0.9)	1.1 (1.9)	5.2 (9.0)	20.4	44.4 [48.1]	0.7 (1.2)	9.9 (11.8)	15.3 (17.9)	19.1 (28.7)
CGHseg	0.5 (0.3)	0.1 (0.3)	0.3 (0.6)	1.5 (2.7)	18.2	29.6 [29.6]	0.4 (0.7)	7.0 (6.4)	7.7 (9.3)	9.6 (12.6)

* Sensitivity of all data, () represents deletion-only data.

Sensitivity: Common data between "other experimental results" and "each CNV detection program's results" divided by "other experimental results".

Commonly utilized individuals between this study and other experiment are used for calculating sensitivity and specificity.

CNV segments with overlap > 80% are regarded as commonly detected segments.

Kidd-validation: Kidd results that are also detected using other experimental methods in the paper^7^.

**Conrad and Shaikh data include only deletion data.

[] represents ratios with overlapping ratio > 30% and CNV length > 10 kb.

The distributions of CNV length are summarized in Figure 1. As shown in Figure 1 (a), the difference of distributions of CNV length between each program is not so large and especially the distribution of CNV length between Birdseye (part of Birdsuite) and PennCNV, which both use HMM, is similar. When Figures 1 (a) and (b) are compared, the lengths of the newly detected CNVs are longer than other experimental results and the total number of detected CNVs are less than others, which are partly caused of used thresholds for detecting CNVs. Shorter CNVs are also detected using these programs before applying thresholds. The distributions of CNV lengths are quite different depending on the CNV experimental method, even if the same individual is used, as shown in Figure 1 (b). Although a gain or loss < 1 kb is not regarded as a CNV but as a deletion or insertion in the DGV criteria [32], all gain and loss data were used as CNVs in this study. When sequencing techniques were used, small gain and loss could be detected, as shown in this figure. On the other hand, when SNP array or array CGH was used, only long CNVs could be detected due to the sparseness of the probe density on a genome.

The results of the section 'Evaluation of CNV detection algorithms using trios data' and 'Evaluation of CNV detection algorithms by comparing other experimental results' indicate that Hidden Markov based programs PennCNV and Birdseye (part of Birdsuite), or Birdsuite (mainly consisting of Birdseye and EM-based Canary) are superior to others when the high CNV reproducible (stability) rates of the same individuals and the low Mendelian inconsistencies are taken into account. For measuring sensitivities with other experimental results, Birdsuite is the best. However, overlapping rates with other experimental results suggest that there remain many false negatives and some false positives, although other experimental results are also expected to contain many false positives and negatives.

### CNVs of original data of healthy Japanese

#### Overlap between DGV, HapMap, and original data

CNVs of the original 180 healthy Japanese individuals are detected by these five programs using parameters that achieved the best performance in HapMap data in Section 'Evaluation of CNV detection algorithms using trios data'. The similarity of CNVs detected using each program is summarized as the sensitivity in supplemental Table 3S [Additional file 2: Supplemental Table 3S]. Table 5 lists the overlap ratios (specificity) of HapMap data and original data with Conrad's data (JPT+CHB 90 individuals) [28] and Park's data (30 Asian individuals) [29] and summarizes the influence of commonality among individuals. Unlike Table 4, the overlap ratio (specificity) of Table 5 was calculated not by individual level, but by whole level (pooled CNVs). Compared to the CNVs detected in the HapMap data, the overlap ratios of the original data with Conrad's data and Park's data are low in all programs except Birdsuite. In every case, Birdsuite, especially the Birdseye module, shows high overlap ratio. When regions commonly detected using at least two programs are selected, the overlap ratio increases except for the results from the Birdseye (part of Birdsuite), as shown in Table 5. Therefore, it is expected that commonly detected CNVs using two programs are reliable CNV regions. When the number of commonly detected individuals increases, the overlap ratio also increases as shown in Table 5, suggesting that the repeatedly detected regions are plausible common CNVs.

**Table 5:** Overlap ratio of original data and HapMap data with Park and Conrad's data

Programs	**Average of overlap ratio of HapMap with Conrad's data**[28]**and Park's data**[29]	**Average of overlap ratio of original data with Conrad's data**[28]**and Park's data**[29]	**Average of overlap ratio of original data of commonly detected regions at least two programs, with Conrad's data**[28]**and Park's data**[29]	**Average of overlap ratio of original data of commonly detected regions in more than two individuals, with Conrad's data**[28]**and Park's data**[29]	**Average of overlap ratio of original data of commonly detected regions in more than nine individuals with Conrad's data**[28]**and Park's data**[29]
PennCNV [8986/5619]	52.2/53.2%*^$^ 44.6/50.8%^$$^	35.8/39.7% 32.1/39.0%	42.5/45.2% 42.7/48.2%	57.8/58.6% 58.6/63.9%	61.8/62.3% 62.9/68.0%
Birdseye (part of Birdsuite) [6959/5063]	68.7/69.6% 62.9/65.6%	71.0/71.6% 81./83.2%	62.1/63.1% 71.4/74.6%	75.6/75.8% 86.7/88.5%	79.4/79.5% 91.6/92.9%
Birdsuite [31852/25794]	66.3/66.6% 53.4/56.5%	65.0/65.3% 63.8/66.8%	65.2/65.9% 73.9/77.1%	67.9/67.9% 67.1/70.1%	70.6/70.6% 69.7/72/7%
DNAcopy [10243/4436]	63.2/64.2% 49.6/54.1%	24.2/27.8% 20.0/25.6%	33.8/36.6% 33.4/38.6%	66.4/66.8% 61.9/66.4%	72.5/72.5% 67.6/71.1%
CGHseg [4043/1966]	54.7/56.6% 38.9/45.4%	31.1/38.8% 21.9/31.7%	38.1/41.6% 33.7/40.4%	68.0/69.6% 56.3/63.3%	74.5/75.5% 60.8/65.5%

*ratio of 80% < CNV overlap segments/ratio of 5% < CNV overlap segments

$:Comparison with Conrad's data, $$ Comparison with Park's data.

[]: represents total number of CNVs/total number of CNV deletions in original data.

The numbers of commonly detected CNVs with HapMap data and original data of healthy Japanese are summarized in supplemental Table 4S [Additional file 2: Supplemental Table 4S]. Since the overlap between original data and HapMap data should be JPT/CHB > CEU > YRI, considering the genetic distance of ethnics, the tendency JPT/CHB > CEU > YRI in any program is reasonable in Table 4S.

#### Characteristics of CNV regions

As interpretations of origin of copy number variations, two mechanisms have been proposed [35]. One is the non-allelic homologous recombination (NAHR, ectopic HR) mediated mechanism and the other is a microhomology-based mechanism. NAHR requires long repeated sequences (up to 300 bp in human [36]) in the start and end regions of CNVs, while microhomology requires only 5-15 bp homology sequences. NAHR is expected to occur by unequal crossing-over and break-induced replication (BIR). Single-strand annealing (SSA) is also a deletion mechanism [35]. In SSA, the complementary single-stranded sequences of the 5'-end of a double-strand break are annealed, and the regions between the two complementary sequences are deleted.

The ratios of the CNV start and end regions where segmental repeats are included, are listed in Table 6. The segmental repeats annotated by UCSC [37] were used. Segmental repeats are sequences that have sequence similarity with another genomic location (> 1kb and > 90% sequence similarity). Although the threshold of repeat length is not imposed in Table 6, the tendencies are not changed even if the threshold is set at 300 bp. The ± 1kbp in Table 6 indicates the regions ± 1kbp of the CNV start and end region, that is, -1k bp to +1k bp of the start region and -1kbp to +1kbp for the end region are used. These comparatively long start and end regions are used because the probes in an array are sparse and both experimental and computational errors are expected in the start and end regions of detected CNVs. Even when the regions are narrowed to ± 500 bp, the tendency of the results is not changed, as shown in Table 6. Common1 represents the CNV regions detected using at least two programs, while Common2 represents the CNV regions detected in at least five individuals by using PennCNV and at least one other program. The enrichment of segmental repeats in CNV regions was reported in previous studies [11,38]. Table 6 shows that the ratios of CNVs including segmental repeats in both start and end regions are higher than those of randomly selected regions. The differences between Common1 and Common2 are not large. Table 6 also lists the ratios of CNVs including interspersed repeats such as long and short interspersed nuclear elements (LINE and SINE, respectively) and long terminal repeat (LTR) in the start and end regions of CNVs. The repeats identified in UCSC [37] using Repeat Masker 3.2.7 were used. The CNV ratios, including repeats in both the start and end regions in Common1 and Common2, are significantly higher than those of randomly selected regions in any interspersed repeat kind except "only SINE". The ratios of Common1 and Common2 are higher than those of randomly selected regions also in "LTR", but the difference is small. Furthermore, the ratios of Common2 are higher than those of Common1 in most cases of any interspersed repeat kind except "only SINE".

**Table 6:** The segmental repeats and interspersed repeats-included percentage of start and end regions of CNV

Programs	Segmental repeats	ALL*	Only SINE	Only LINE	Only LTR	Segmental repeat +interspersed repeats (ALL*)
Random* ± 500bp	2.8	17.3*	0.0	7.8	1.6	19.6
Random ± 1kbp	2.9	21.7	0.4	10.6	1.9	24.1
CNVCommon1 ± 500bp	34.0	24.8	0.0	12.4	2.3	53.9
CNVCommon1 ± 1kbp	34.6	39.5	1.3	21.9	5.6	62.7
CNVCommon2 ± 500bp	32.2	29.1	0.0	11.6	2.5	53.7
CNVCommon2 ± 1kbp	33.1	43.8	0.0	25.8	5.0	65.3

ALL* means all interspersed repeats of repeatmasker 3.2.7.

CNVcommon1: 315 CNV regions detected by at least two programs and more than once.

CNVcommon2: 121 CNV regions detected by PennCNV more than four times (that is, at least five individuals) and at least one other program. Random*:1000 randomly extracted regions whose lengths are close to extracted CNV lengths.

In all detection programs, with an increasing number of commonly detected individuals, the CNV ratios containing interspersed repeats or segmental repeats also increased as shown in supplemental Table 5S [Additional file 2: Supplemental Table 5S]. (It should be noted that there are no obvious relationships between these ratios and the stability rate or the Mendelian inconsistency ratio of each program.) When the ratios of CNVs including "segmental repeats or interspersed repeats" in start and end regions of CNVs are calculated, the results are higher than "segmental repeats" only or "interspersed repeats (ALL)" only as shown in Table 6. About 40% of segmental repeats detected around CNVs include interspersed repeats. Of these segmental repeats, LINE and LTR are about 50% and 15%, respectively. Since LINE, SINE, and LTR account for about 52%, 14%, 23% of all interspersed repeats in the human genome (hg18), respectively, there are no LINE biases in the segmental repeats around CNVs. The enrichment of interspersed repeats and segmental repeats is also observed in previous experimental data as shown in supplemental Table 6S [Additional file 2: Supplemental Table 6S]with a few exceptions.

These results indicate that not only segmental repeats but also interspersed repeat regions, especially "LINEs", have an important role in the formation of CNVs, at least in frequently observed copy number variations, although there is a possibility that interspersed repeats promote segmental repeats. Although both SINE and LINE elements have been reported to contribute structural variations [39], our results and those of others [11] do not support a contribution of SINE to formation of CNVs.

When the CNV ratios including simple repeats and mobile elements (provided in UCSC) in both the start and end regions are compared, there are no differences among Common1, Common2, and random values. The recombination rate of these regions (± 500 bp) calculated using the Marshfield average does not show statistically meaningful differences among random values ( = 1.17) and CNV common1 ( = 1.14) and common2 ( = 1.18).

The expected length, 5-15 bp, of microhomology is too short to use array-based CNV detection methods. In many CNV start and end regions, microhomologous sequences were found but there were almost no statistical significances (for example, 4bases^5bp is at most 1024) when the start and end positions of CNVs were ambiguous due to sparseness of probe positions. Conrad *et al. *[40] have investigated breakpoints of CNV deletions by massive parallel sequencer and found 70% of them have microhomology. Sequencing of these regions is necessary to confirm their importance.

Table 7 compares the conservation scores from primates (UCSC phastCons44wayPrimates [37]). This table indicates that the start and end regions of CNVs are lower than random values, and CNV Common2 shows smaller conservation scores than those of CNV Common1. In all detection methods, as the number of commonly detected individuals increased, the average conservation score of the CNV start and end regions decreased (data not shown). When the number of CpG islands is counted in the CNV start and end regions, it is slightly smaller than those in random regions (P = 0.034). This seems reasonable because CpG islands are sparse in repeat regions.

**Table 7:** Average conservation score of start and end of CNV regions

Regions	Average conservation score in start regions	Average conservation score in end regions
Random ± 500bp	62.7	67.8
Random ± 800bp	60.9	77.0
Random ± 1kbp	69.1	76.0
CNVCommon1 ± 500bp	38.2	51.1
CNVCommon1 ± 800bp	42.5	55.6
CNVCommon1 ± 1kbp	47.9	62.3
CNVCommon2 ± 500bp	32.9	47.6
CNVCommon2 ± 800bp	35.7	50.8
CNVCommon2 ± 1kbp	39.4	57.0

CNVcommon1: 315 CNV regions detected by at least two programs and more than once.

CNVcommon2: 121 CNV regions detected by PennCNV more than four times and at least one other program

Scored sum value of phastCons44wayPrimates (UCSC) is used as Conservation score.

...

The above-mentioned tendencies in the start and end regions of CNVs were also observed in our CNV results of HapMap data, and differences between CNV duplications and deletions were not observed. It should be noted that although the overlapping ratio between our CNV results of HapMap data and other experimental data are low, most of them have the similar tendency that both segmental repeats and interspersed repeats (especially LINE) are enriched in CNV start and end regions, and conservation scores based on primates are lower in these regions than in randomly selected regions.

#### CNV database construction

In the Japanese Integrated Database Project of MEXT, our organization (Univ. of Tokyo, Univ. of Tokai, and Hitachi, Ltd.) has constructed and maintained a public repository for the genome-wide association study (GWAS) database for continuous and intensive management of GWAS data and to facilitate data-sharing among researchers [41]. A CNV control database has been constructed as a part of the GWAS database.

**Figure 3:**
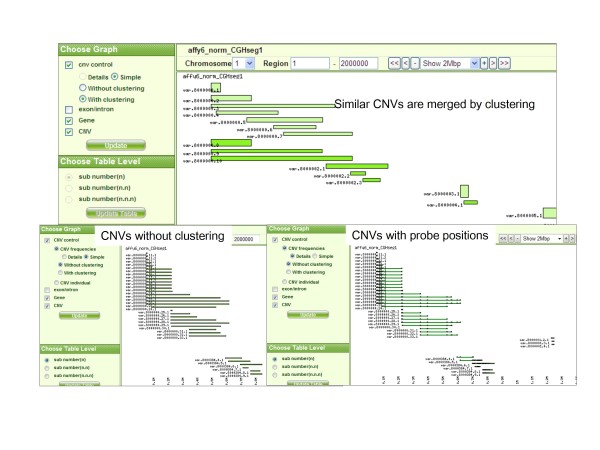
**Screen shots of CNV region information in CNV control database**. Screenshot of CNV region information with/without clustering and with/without information for each probe in CNV control database.

Since the start and end positions are slightly different between individuals, this makes it difficult to understand common CNV structures. Accordingly, CNVs with start and end positions located within 20 probes are regarded as the same CNV regions by clustering CNVs of all individuals. Screenshots of the CNV control database are shown in Figures 2 and 3. As shown in Figure 2, interfaces for the overview of CNVs on whole genomes and for region information are provided. Results both with and without clustering can be depicted, as shown in Figure 3. The above-mentioned original data are stored in this database.

In this system, bulk CNV data are freely available, but a simple application is required for accessing CNV data at the individual level. To use raw data for CNVs, researchers must submit an application describing the research purpose in detail to the database access committee. Access is determined at the data sharing review board. The details of the data sharing policy are summarized in http://gwas.lifesciencedb.jp/gwasdb/db_policy_en.html.

The CNV control database is accessible at http://gwas.lifesciencedb.jp/cgi-bin/cnvdb/cnv_top.cgi.

## Conclusions

In this study, we evaluated five widely used CNV detection programs, Birdsuite, Birdseye (part of Birdsuite), PennCNV, CGHseg, and DNAcopy from the viewpoint of performance on the Affymetrix platform using HapMap data and other experimental data. Our results indicate that hidden Markov based programs PennCNV and Birdseye (part of Birdsuite), or Birdsuite are superior to others when the high CNV detection stability (reproducibility) rates of the same individuals and the low Mendelian inconsistencies are considered. For measuring the sensitivity of other experimental results, Birdsuite shows the best performance. However, the low overlapping rates with other experimental results imply that there remain many false negatives and some false positives, although other experimental results also contain many false positives and negatives.

The analysis of start and end regions of CNVs in the data for healthy Japanese and the HapMap data showed that both segmental repeats and interspersed repeats are enriched in CNV start and end regions, suggesting that not only segmental repeats but also interspersed repeats, especially LINE, are deeply involved in CNV formation, particularly in common CNV formations, although the previous studies mainly focused on segmental repeats [9,11]. There are CNVs without segmental repeats or interspersed repeats. They might contain microhomologies or other characteristics, the resolution of SNP array seems too coarse to analyze microhomologies. Other sequence level technologies will be required for further detailed analysis.

## Authors' contributions

KT participated in the conception and design of the study and interpreted the results. NN and DY designed and coordinated the experiment. AK participated in the conception and design of the study, carried out the analysis, and wrote the manuscript. All authors reviewed analysis results and read and approved the final manuscript.

## Supplementary Material

Additional file 1 **Supplementary Figures**. Figure 1S (a) Relationship between standard deviation of probe intensity log_2_ratio of each microarray and number of CNV segments per individual in HapMap data. (b) Relationship between call rate (the percentage of probes with genotypes determined in the genotype calling process) and number of CNV segments per individual in HapMap data. Figure 2S (a) Relationship between standard deviation of probe intensity log_2_ratio of each microarray and number of CNV segments per individual in original data. (b) Relationship between call rate and number of CNV segments per individual in original data. Click here for file

Additional file 2 **Supplementary Tables**. Click here for file
