# Supplementary material for: Comparative analysis of copy number variation detection methods and database construction
Source: BMC Genet. 2011 Mar 7;12:29. doi: 10.1186/1471-2156-12-29 (PMC3058066; doi:10.1186/1471-2156-12-29)
Supplement: Additional file 1 — Supplementary Figures. Figure 1S (a) Relationship between standard deviation of probe intensity log2ratio of each microarray and number of CNV segments per individual in HapMap data. (b) Relationship between call rate (the percentage of probes with genotypes determined in the genotype calling process) and number of CNV segments per individual in HapMap data. Figure 2S (a) Relationship between standard deviation of probe intensity log2ratio of each microarray and number of CNV segments per individual in original data. (b) Relationship between call rate and number of CNV segments per individual in original data. [file 1471-2156-12-29-S1.PPT]

## Slide 1
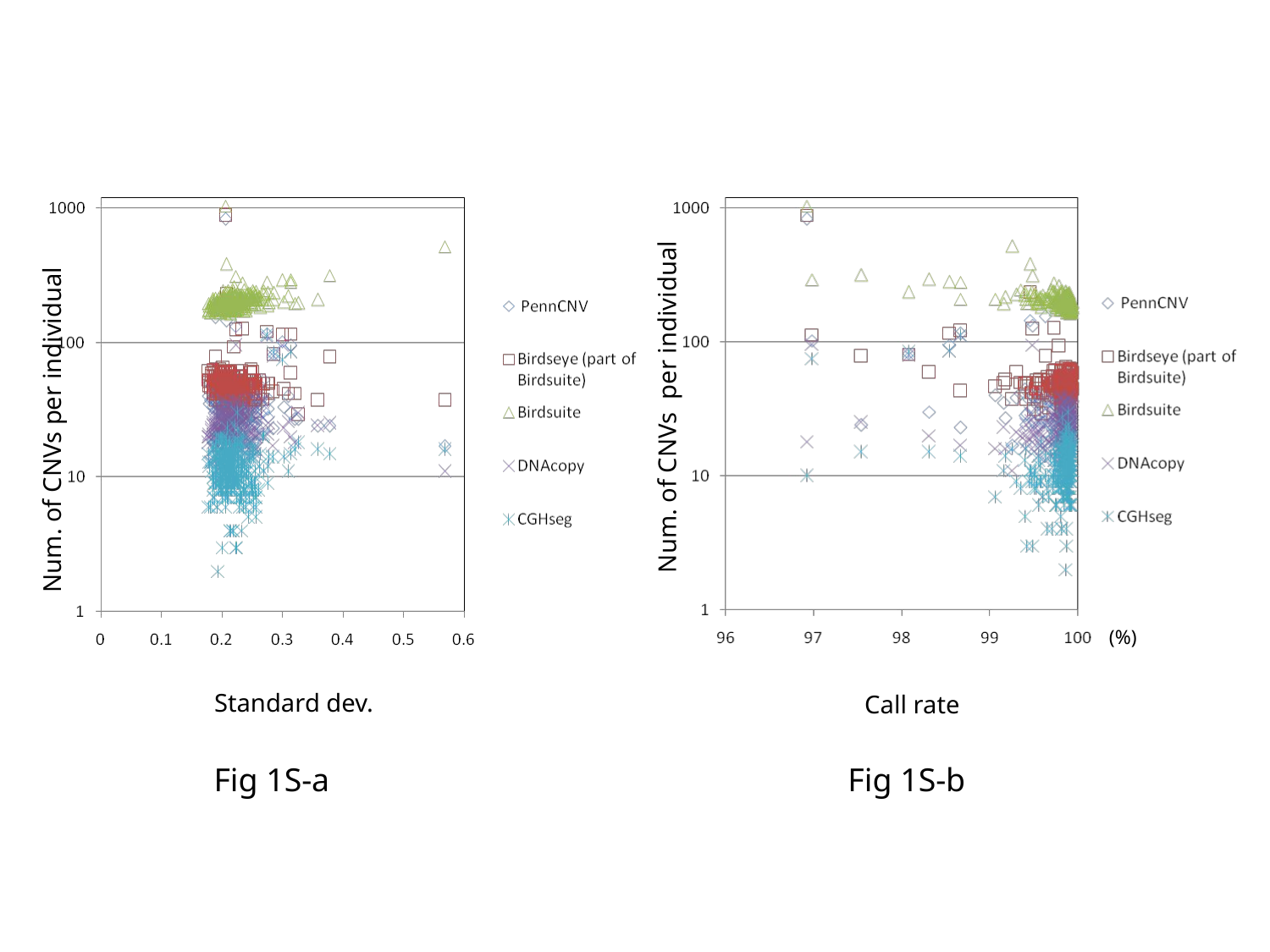

Num. of CNVs per individual
Num. of CNVs per individual
(%)
Standard dev.
Call rate
Fig 1S-a
Fig 1S-b

## Slide 2
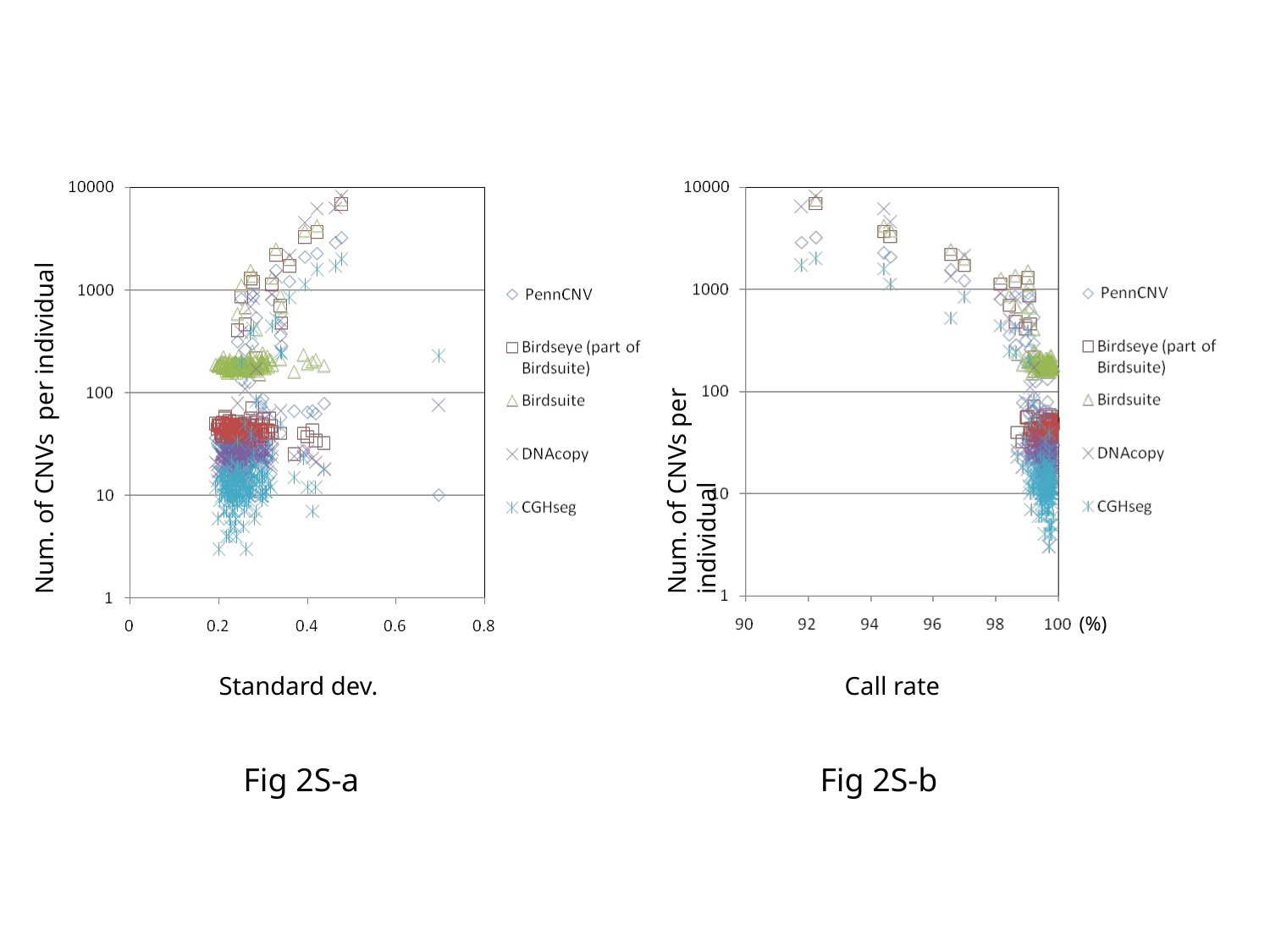

Num. of CNVs per individual
Num. of CNVs per individual
(%)
Standard dev.
Call rate
Fig 2S-a
Fig 2S-b
