# Supplementary material for: Comparative analysis of copy number variation detection methods and database construction
Source: BMC Genet. 2011 Mar 7;12:29. doi: 10.1186/1471-2156-12-29 (PMC3058066; doi:10.1186/1471-2156-12-29)
Supplement: Additional file 2 — Supplementary Tables. [file 1471-2156-12-29-S2.DOC]

Supplemental

Table1S Specificity of each detection program when other experimental result is regarded as golden standard

| Programs | Korbel^[6]^  (PE sequencing) | Kidd^[7]^  (PE sequencing) | Kidd^[7]^  -validation | Tuzun^[8]^  (PE sequencing) | Conrad^[25]**^  (Mendelian consistency) | Shaikh^[26]^**  (Illumine Data) | Redon^[11]^  (Tiling array) | Perry^[27]^  (array CGH) | Conrad^[28]^  (Tiling array) | Park^[29]^  (array-CGH  and massively parallel sequencing) |
| --- | --- | --- | --- | --- | --- | --- | --- | --- | --- | --- |
| PennCNV | 40.3*  (44.1) | 41.6  (63.6) | 39.6  (60.5) | 38.1  (62.7) | 6.5 | 11.1  [33.3] | 16.3  (13.8) | 75.6  (62.8) | 44.6  (59.8) | 53.4  (62.8) |
| Birdseye (part of Birdsuite)  Birdsuite | 42.7  (43.6)  34.0  (35.0) | 51.9  (66.9)  37.9  (45.6) | 50.2  (64.5)  36.8  (44.2) | 48.7  (62.4)  36.1  (40.9) | 5.1  2.2 | 10.9  [28.3]  7.9  [16.4] | 12.9  (8.8)  6.1  (4.6) | 76.3  (74.3)  40.0  (36.8) | 68.7  (73.8)  45.4  (46.9) | 71.4  (74.3)  40.2  (38.6) |
| DNAcopy | 45.5  (50.0) | 45.8  (61.6) | 43.3  (58.2) | 37.3  (52.7) | 5.4 | 18.2  [45.5] | 20.1  (17.5) | 72.6  (70.8) | 58.6  (69.3) | 69.9  (77.1) |
| CGHseg | 66.7  (81.8) | 36.5  (51.5) | 35.4  (50.0) | 24.3  (50.0) | 9.9 | 35.0  [55.0] | 22.2  (17.9) | 72.2  (68.4) | 51.7  (63.2) | 63.7  (65.5) |

* Specificity of all data, () represents deletion-only data.

Specificity: Common data between “other experimental results” and “each CNV detection program’s results” divided by “CNV detection program’s results”.

Commonly utilized individuals between this study and other experiment are used for calculating sensitivity and specificity.

CNV segments with overlap > 80% are regarded as commonly detected segments.

Kidd-validation: Kidd results that are also detected using other experimental methods in the paper^7^.

**Conrad and Shaikh data include only deletion data.

[] represents ratios with overlapping ratio > 30% and CNV length > 10 kb.

Table 2S Sensitivity of each experiment when other experimental result is regarded as golden standard

| Experiments  (golden standard)  Experiments | Korbel  (PE sequencing) | Kidd  (PE sequencing) | Tuzun  (PE sequencing) | Conrad*  (Mendelian consistency) | Shaikh*  (Illumine Data) | Redon  (Tiling path array) | Perry  (array CGH) | Conrad  (Tiling array) | Park  (array-CGH  and sequencing) |
| --- | --- | --- | --- | --- | --- | --- | --- | --- | --- |
| Korbel | - | 10.5/15.6% (27/16) | 11.1/15.2% (22/15) | - | - | 10.3/28.2% (24/11) | - | - | - |
| Kidd | 9.0/12.0% (32/32) | - | 87.1/91.9% (202/91) | 40.0/40.0% (20/20) | 70.4/70.4% (19/19) | 0.2/0.6%  (1/1) | 7.3/13.9%  (96/83) | 5.6/6.0%  (723/715) | - |
| Tuzun | 9.3/12.5%  (32/32) | 77.3/90.0%  (197/90) | - | - | - | - | - | - | - |
| Conrad* | - | 0.1/0.2%  (1/1) | - | - | 20/20%  (4/4) | 0.1/0.3% (2/2) | 1.2/2.4%  (39/39) | 0.1/0.2%  (60/60) | - |
| Shaikh* | - | 0.3/0.9%  (2/2) | - | 18.2/18.2%  (2/2) | - | 0.0/0.0% (0/0) | - | 0.3/0.4%  (14/14) | - |
| Redon | 1.3/1.0%  (8/4) | 1.3/1.8%  (103/39) | - | 6.8/8.3% (58/58) | 0.0/0.0%  (0/0) | - | 13.4%(807) | 1.0/0.7% (3481/1494) | 3.0/1.9% (303/105) |
| Perry | - | 2.5/3.5%  (33/12) | - | 24.3/24.3% (46/46) | - | 6.3/8.8% (108/66) | - | 2.3/2.2%  (924/741) | 13.9/14.0%  (452/344) |
| Conrad (tiling) | - | 3.3/5.8%  (136/124) | - | 21.1/21.0%  (72/72) | 88.9/88.9%  (24/24) | 8.3/5.1%  (405/110) | 17.5/25.4%  (896/636) | - | - |
| Park | - | - | - | - | - | 1.4/4.7%  (13/2) | 13.2/17.5%  (370/254) | - | - |

Each value represents the sensitivity of experiment (deletion+duplication/deletion) in column when other experimental result in row is regarded as golden standard.

Commonly utilized individuals between experiments are used for calculating sensitivity.

“-“ :two experiments do not include common individuals.

CNV segments with overlap > 80% are regarded as commonly detected segments.

():represents actual CNV counts (deletion+duplication/ deletion)

*Conrad and Shaikh data include only deletion data.

Supplemental

Table 3S Similarity (sensitivity) of each method using overlap data of original data

| Programs | PennCNV | Birdseye (part of Birdsuite)  Birdsuite | DNAcopy | CGHseg |
| --- | --- | --- | --- | --- |
| PennCNV [8675/5391] | - | 31.5/45.7%  (36.8/48.8%)  15.4/18.0%  (16.5/19.0%) | 46.5/57.1%  (32.8/47.9%) | 37.8/60.0%  (30.5/48.9%) |
| Birdseye (part of Birdsuite) [6959/5063]  Birdsuite [30012/23954] | 30.9/35.6%  (40.5/45.2%)  34.9/39.1%  (44.9/49.1%) | - | 15.4/19.1%  (29.9/36.0%)  20.1/22.7%  (39.0/43.2%) | 17.3/23.7%  (25.0/32.5%)  22.7/27.8%  (33.7/39.5%) |
| DNAcopy [10243/4436] | 39.8/50.9%  (36.8/42.0%) | 21.8/28.5%  (10.2/31.7%)  11.9/11.0%  (13.1/12.1%) | - | 53.0/75.7%  (59.5/69.8%) |
| CGHseg  [4043/1966] | 29.9/32.9%  (20.7/23.5%) | 11.5/13.1%  (10.2/11.9%)  4.0/4.9%  (3.7/4.7%) | 51.6/53.6%  (37.1/40.3%) | - |

*80%overlap/5%overlap of CNV segments.

Sensitivity: percentage of CNVs detected using the program in row, which is also detected using the program in column. That is, each program in row is regard as golden standard.

(): represents deletion data only, []: represents total number of CNVs/total number of CNV deletions.

Supplemental

Table 4S Overlap ratio of original data with HapMap calculated by each program

| Programs | JPT or CHB | CEU | YRI | Non-overlap with HapMap |
| --- | --- | --- | --- | --- |
| PennCNV | 34.3/36.5%* | 28.9/30.7% | 26.0/27.8% | 63.1/60.2% |
| Birdseye (part of Birdsuite)  Birdsuite | 42.9/42.4%  51.1/51.1% | 37.5/38.0%  47.4/47.6% | 31.1/32.1%  46.1/46.2% | 55.5/53.0%  47.7/47.7% |
| DNAcopy | 38.3/40.2% | 35.0/36.6% | 32.3/34.0% | 55.8/58.4% |
| CGHseg | 28.1/30.3% | 23.2/26.1% | 22.0/25.8% | 67.4/64.3% |

*80%overlap/5%overlap of CNV segments.

Supplemental

Table 5S Segmental repeats and interspersed repeats included-ratio of start and end regions of CNVs

| Programs and regions | Segmental repeats | ALL* | Only SINE | Only LINE | Only LTR | Segmental repeat  +interspersed repeats (ALL*) |
| --- | --- | --- | --- | --- | --- | --- |
| Random** ±1kbp | 2.9 | 21.7 | 0.4 | 10.6 | 1.9 | 24.1 |
| PennCNV (>1) ±1kbp | 13.9 | 27.6 | 1.0 | 11.9 | 2.2 | 38.9 |
| PennCNV (>5) ±1kb | 32.0 | 40.5 | 0.0 | 22.9 | 5.2 | 62.7 |
| Birdseye (part of Birdsuite)  (>1) ±1kbp | 26.4 | 28.4 | 0.9 | 13.4 | 3.3 | 48.0 |
| Birdseye (part of Birdsuite)  (>5) ±1kbp | 40.7 | 39.0 | 1.1 | 20.3 | 3.3 | 65.4 |
| DNAcopy(>1) ±1kbp | 16.7 | 28.7 | 0.4 | 18.2 | 4.1 | 46.0 |
| DNAcopy(>5) ±1kbp | 25.0 | 44.0 | 0.0 | 31.9 | 6.9 | 68.1 |
| CGHseg(>1) ±1kbp | 20.3 | 26.9 | 0.3 | 15.3 | 4.2 | 49.7 |
| CGHseg(>5) ±1kbp | 27.8 | 34.3 | 0.0 | 50.0 | 0.0 | 72.2 |

ALL*: means all interspersed repeats of repeatmasker 3.2.7.

Random**:1000 randomly extracted regions with lengths close to the extracted CNV lengths.

(>1): CNV regions commonly detected more than one individual.

(>5): CNV regions commonly detected by more than five individuals.

Supplemental

Table 6S Segmental repeats and interspersed repeats included-ratio of start and end regions of CNVs in various experiments.

| Experiments | Segmental repeats | ALL* | Only SINE | Only LINE | Only LTR | Segmental repeat  +interspersed repeats (ALL*) |
| --- | --- | --- | --- | --- | --- | --- |
| Korbel | 22.4 | 35.7 | 0.8 | 17.4 | 5.4 | 51.8 |
| Kidd | 8.0 | 27.1 | 0.7 | 12.4 | 2.6 | 34.6 |
| Kidd-validation | 16.5 | 28.9 | 1.1 | 13.3 | 3.3 | 44.0 |
| Tuzun | 24.1 | 27.0 | 2.9 | 12.0 | 3.7 | 47.7 |
| Conrad** | 8.7 | 33.0 | 0.6 | 15.8 | 5.3 | 40.5 |
| Shaikh** | 0.0 | 44.4 | 0.0 | 22.2 | 0.0 | 44.4 |
| Redon | 16.8 | 28.2 | 0.2 | 12.3 | 4.0 | 49.4 |
| Perry | 48.2 | 27.7 | 1.6 | 12.5 | 3.8 | 67.8 |
| Conrad (tiling) | 13.7 | 37.7 | 2.5 | 23.3 | 7.4 | 51.3 |
| Park | 10.3 | 42..3 | 2.7 | 16.1 | 5.6 | 42.4 |

ALL*: means all interspersed repeats of repeatmasker 3.2.7.

**Conrad and Shaikh data include only deletion data.

±1kbp of the start and end regions of CNVs are used.
